# Cyclophotocoagulation under Microscopy Combined with Phacoemulsification for Primary Angle-Closure Glaucoma

**DOI:** 10.1155/2021/6915656

**Published:** 2021-10-21

**Authors:** Xiaoli Xiang, Haiying Chen, Jian Li, Pan Xiao, Wei Zhu, Ling Miao, Zhengru Huang

**Affiliations:** Department of Ophthalmology, The Affiliated Changshu Hospital of Xuzhou Medical University, Changshu, China

## Abstract

The purpose of this study was to evaluate the safety and efficacy of cyclophotocoagulation under microscopy combined with phacoemulsification in patients with primary chronic angle-closure glaucoma. We retrospectively reviewed the results of cyclophotocoagulation under microscopic direct vision combined with phacoemulsification in 35 eyes (35 patients) with primary chronic angle-closure glaucoma and coexisting visually significant cataracts, treated between January 2017 and April 2020 at the glaucoma unit of the affiliated Changshu Hospital of Xuzhou Medical University. All patients were followed up for at least 12 months postoperatively. The preoperative to postoperative changes in best-corrected visual acuity (BCVA), intraocular pressure (IOP), number of antiglaucoma medications, and surgery-associated complications were recorded. The BCVA improved from 1.15 ± 0.91 logMAR preoperatively to 0.86 ± 0.82 logMAR at the final postoperative examination (*Z* = −3.62, *P* < 0.0001). The mean IOP was 36.63 ± 13.50 mmHg preoperatively and 15.14 ± 3.19 mmHg at the final examination (*Z* = −5.16, *P* < 0.0001). The number of antiglaucoma drugs was significantly reduced from 2.23 ± 0.55 preoperatively to 0.54 ± 0.86 at the final postoperative examination (*Z* = −5.26, *P* < 0.0001). The absolute value of the mean defect and retinal nerve fiber layer thickness at the last follow-up postoperatively were significantly reduced compared to preoperative values (*Z* = −3.35, *P*=0.001; *Z* = −4.56, *P* < 0.001, respectively). One patient experienced an explosive suprachoroidal hemorrhage during the operation. The sclera was incised at the corresponding site of the intraoperative hemorrhage. The operation was continued once there was no active bleeding, and the outcome was satisfactory. None of the patients required additional surgery to treat complications. Thus, cyclophotocoagulation under microscopic direct vision combined with phacoemulsification can be performed safely for the management of primary angle-closure glaucoma.

## 1. Introduction

Primary angle-closure glaucoma (PACG) is a type of glaucoma estimated to affect approximately 26% of the glaucoma population; however, PACG is responsible for nearly half of the cases of glaucoma-related blindness worldwide [1]. In 2014, 20 million people worldwide were diagnosed with PACG, and this number is expected to increase to 34 million by 2040. East Asia, including China, has the highest prevalence of PACG [2,3]. The effective treatment of PACG and avoidance of blindness caused by PACG are major challenges in clinical studies of glaucoma.

Different types of lasers have been widely used to treat different types of glaucoma. Endoscopic ciliary body photocoagulation (ECP) is considered to be a promising approach for the treatment of various types of glaucoma, including PACG, and may become the treatment of choice [4]. However, ECP requires expensive specialized 810 nm laser instruments and endoscopic monitoring and display equipment, which limits the promotion and application of ECP in developing regions. Accordingly, it is necessary to develop a simple, effective, and convenient method for photocoagulation of intraocular ciliary processes based on ECP.

Therefore, we aimed to evaluate the safety and efficacy of cyclophotocoagulation under microscopic direct vision combined with phacoemulsification in patients with PACG.

## 2. Materials and Methods

This study was approved by the institutional review board of Changshu Hospital of Xuzhou Medical University (Changshu, China) (approval number: 2016034). This study was conducted in accordance with the Declaration of Helsinki. Informed consent for the surgery and participation in this study was obtained from all patients.

### 2.1. Patients

We conducted a retrospective case review of consecutive patients with refractory glaucoma treated between January 2017 and April 2020 at the glaucoma unit of the affiliated Changshu Hospital of Xuzhou Medical University (Changshu, China) who underwent cyclophotocoagulation under direct microscopic vision. The inclusion criteria were as follows: PACG, intraocular pressure (IOP) not controlled by confirmed medical treatment (IOP > 21 mmHg despite maximally tolerable medication use), visual field tubular or temporal island in nature, *C*/*D* > 0.8, and complicated cataracts requiring surgery.

The exclusion criteria were as follows: drug therapy could control the IOP to ≤21 mmHg, complicated with cataracts of varying degrees not requiring surgery, best-corrected visual acuity (BCVA) > 20/40, pupil could not be dilated preoperatively, combined with retinal diseases such as diabetic retinopathy or retinal vein occlusion, preoperative B-type ultrasound examination showed severe vitreoretinal traction or retinal detachment, accompanied by severe systemic disease or mental illness, and inability to tolerate surgery.

### 2.2. Assessments

Slit-lamp microscopy, fundus examination, A/B ultrasound, ultrasonic biological microscopy, Humphrey field of vision assessment (Carl Zeiss, Germany), retinal nerve fiber layer thickness (RNFLT) (Cirrus HD-OCT5000, Carl Zeiss, Germany), and corneal endothelioscopy were completed in all patients preoperatively. Additionally, the BCVA, IOP, mean defect (MD), RNFLT, intraoperative complications, photocoagulation treatment range, postoperative complications, time of occurrence, and the type and quantity of IOP-lowering drugs used preoperatively and postoperatively were recorded. The BCVA was measured using a standard logarithmic visual acuity chart, recorded in decimal form and converted to logMAR for statistical analysis (LogMAR = log[1/VA]) [5].

### 2.3. Surgical Procedures

**Figure 1:**
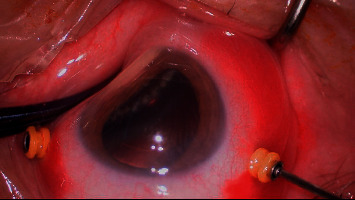
Laser fibers were inserted, and ciliary processes, with the septum, were lasered, one by one, under direct vision.

Surgery was performed under peribulbar anesthesia for all patients. A 23G vitrectomy cannula system was placed 4 mm above the nose and 4 mm behind the corneal limbus, above the temporal. A 2.8 mm transparent corneal tunnel incision on the upper right and a transparent corneal puncture incision on the upper left were made. Sodium hyaluronate was injected into the anterior chamber, followed by phacoemulsification, to remove the lens nucleus, and injection and aspiration of the lens cortex after continuous annular capsulotomy. The corneal incision was watertight. Part of the anterior vitreous was removed (CONSTELLATION® Vision System, Alcon, USA), and the ciliary processes were exposed to the pupil area by pushing against the contralateral eye wall. Laser fibers were inserted through the supranasal and supratemporal incisions, and ciliary processes, with the septum, were lasered, one by one, under direct vision (VISULAS 532s fundus laser therapeutic instrument, Carl Zeiss, Germany). The degree of photocoagulation was as follows: laser pulse time, 300 ms; energy, 200 mW. The photocoagulation reaction was controlled by adjusting the distance between the laser fiber and the ciliary processes (Figure 1). The ciliary processes became contracted and white, but without bubbles, as is standard. The photocoagulation range was based on the preoperative IOP as follows: 21–40 mmHg, 180°; 41–50 mmHg, 210°; and >50 mmHg, 270° [6–8]. The intraocular lens was implanted into the capsule after photocoagulation of the ciliary processes, if necessary.

Tobramycin, dexamethasone eye drops (TobraDex, Alcon-Couvreur SA N.V., Belgium), nonsteroidal anti-inflammatory eye drops (Pranolulin, Senju Pharmaceutical Co., Ltd., Fukusaki Plant, Japan), and tropicamide phenylephrine eye drops (Santen Pharmaceutical Co., Ltd., Shiga Plant, Japan) were administered four times daily for 4–6 weeks postoperatively. Antiglaucoma eye drops were administered if the IOP was >21 mmHg. If the IOP was ≤21 mmHg, but visual field or RNFL damage was aggravating, antiglaucoma medications were also used. The patients were followed up for at least 12 months.

### 2.4. Statistical Analysis

Data are reported as the mean ± standard deviation or number (percentage). For quantitative variables, histograms, box graphs, and Shapiro–Wilk tests were used to evaluate the normality of the distribution. If the data did not fit the normal distribution, nonparametric tests were used. Kaplan–Meier survival analysis of the success rate of the operation was performed (criteria for success: 8 ≤ postoperative IOP ≤ 20 mmHg, without drug treatment). Statistical analyses were performed using SPSS 19.0 statistical software (SPSS Inc., Chicago, IL, USA) and GraphPad Prism 5.0 (GraphPad Software Inc., San Diego, California, USA), and *P* values <0.05 were considered significant.

## 3. Results

### 3.1. Baseline Characteristics

In total, 35 eyes from 35 patients with PACG were included in this study. The average age was 69.14 ± 6.80 years (range, 56–86 years) and 16 patients (45.71%) were female.

### 3.2. Number of Antiglaucoma Drugs

Twelve months after surgery, the IOP was maintained below 20 and 15 mmHg in 28 and 21 patients, respectively, without any antiglaucoma drugs. Considering the target IOP, we used antiglaucoma drugs therapy in seven patients whose IOP were ≤21 mmHg, but visual field or RNFL damage was aggravating. The IOP was controlled below 15 mmHg in 10 and four patients using one and two antiglaucoma drugs postoperatively, respectively. The number of antiglaucoma drugs was significantly reduced from 2.23 ± 0.55 preoperatively to 0.54 ± 0.86 at the final postoperative examination (*Z* = −5.26, *P* < 0.0001).

### 3.3. BCVA

Additionally, the BCVA was significantly improved from 1.15 ± 0.91 logMAR preoperatively to 0.86 ± 0.82 logMAR at the final examination (*Z* = −3.62, *P* < 0.0001).

### 3.4. IOP

**Figure 2:**
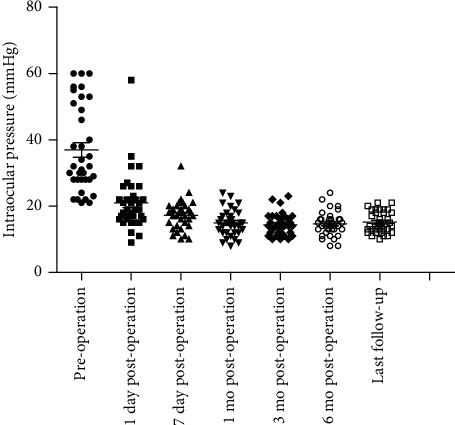
Scatter gram of intraocular pressure before and after cyclophotocoagulation, with postoperative assessments at 1 day, 7 days, 1 month, 6 months, and the last follow-up.

The mean IOP was 36.63 ± 13.50 mmHg preoperatively and 21.06 ± 8.68 mmHg on the first day postoperatively, with a higher IOP than the preoperative IOP in four eyes. The mean IOP was 17.26 ± 4.35 mmHg at 7 days postoperatively, with a lower IOP than the preoperative IOP in all eyes. The mean IOP was 14.94 ± 3.92 mmHg at 1 month postoperatively, with a higher IOP than the preoperative IOP in one eye. The mean IOP was 14.40 ± 3.31 mmHg at 3 months postoperatively, 14.63 ± 3.60 mmHg at 6 months postoperatively, and 15.14 ± 3.19 mmHg at the final examination (Figure 2). Compared to the preoperative IOP level, the postoperative IOP was significantly decreased at each postoperative time point (*Z* = −4.62, −5.16, −5.14, −5.09, −5.14, −5.16; *P* < 0.0001, respectively). However, there were no significant differences in IOP among postoperative time points.

**Figure 3:**
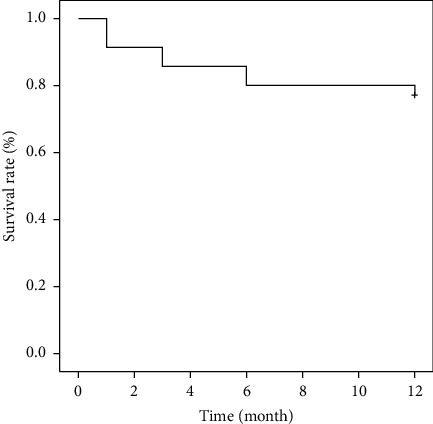
Kaplan–Meier survival curve for success after cyclophotocoagulation under microscopic direct vision combined with the phacoemulsification for primary angle-closure glaucoma. Success was defined as 8 ≤ postoperative intraocular pressure ≤20 mmHg without medications.

The Kaplan–Meier survival curve is shown in Figure 3. The success rate was 80% at 6 months and 77.1% at 1 year postoperatively.

### 3.5. Photocoagulation Range

Eleven patients (31.4%) had a photocoagulation range of 180°, 11 patients (31.4%) had a photocoagulation range of 210°, two patients had a photocoagulation range of 225° (5.7%), five patients (14.3%) had a photocoagulation range of 240°, and six patients (17.1%) had a photocoagulation range of 270°. The average photocoagulation range was 215.86 ± 32.14°, and median value was 210° (range, 180–270°).

There was no linear correlation between the photocoagulation range and the preoperative to postoperative change in IOP at the final examination (*r* = 0.189, *P*=0.278).

### 3.6. MD

The absolute value of the MD at last follow-up postoperatively was significantly reduced compared to the preoperative values (*Z* = −3.35, *P*=0.001) (Table 1).

### 3.7. RNFLT

The RNFLT at the last follow-up postoperatively was significantly reduced from preoperative values (*Z* = −4.56, *P* < 0.001) (Table 1).

### 3.8. Complications

During surgery, a 69-year-old woman experienced an explosive suprachoroidal hemorrhage. A radial scleral incision was made at the corresponding site (temporal side), and the surgery was completed once there was no active bleeding. The range of photocoagulation was 210°. At the final examination, her IOP was 13 mmHg and BCVA was 20/40.

Fibrinous exudation occurred postoperatively in four patients, which was absorbed within 1 month after the topical application of TobraDex eye drops and eye ointment.

On the first day postoperatively, the IOP was >21 mmHg in 14 patients. For 12 of these patients, the IOP was <21 mmHg at 2–3 days after treatment with carteolol hydrochloride eye drops (Mikelan, China Otsuka Pharmaceutical Co., Ltd.), and the IOP was stable after the withdrawal of medication. The IOP of the other two patients was controlled to <21 mmHg at 1 week after treatment with Mikelan and brinzolamide ophthalmic suspension (AZOPT, Alcon-Couvreur SA), and the IOP was stable after the withdrawal of medication.

None of the patients required additional surgery to treat complications.

## 4. Discussion

In the past, ciliary process photocoagulation was mainly used to control the IOP and relieve pain in patients with end-stage glaucoma who had visual function loss [9]. ECP was first applied to reduce the complications of transscleral ciliary process photocoagulation in 1992 [10]. ECP has been shown to be effective in reducing the IOP and is safe for the treatment of refractory and uncontrolled glaucoma. Additionally, ECP has been used as a safe and effective treatment for patients with mild-to-moderate glaucoma [11]. ECP has attracted increasing attention in the treatment of glaucoma and can be used in combination with cataract surgery as the preferred treatment for many types of glaucoma, with fewer complications than traditional trabeculectomy [4,12–14].

Standard ECP photocoagulates the first 2/3 of each ciliary process through the anterior chamber and pupil via a clear corneal incision and cannot photocoagulate the septum of the ciliary process [12,14]. Although one incision can achieve a range of 270°, and two incisions can achieve 360°, in ciliary process photocoagulation, the reduction in IOP by this anterior-approach ECP is still limited [14,15]. However, as ECP-plus is performed close to the ciliary process via a flat puncture incision, the whole ciliary process, with the septum, can be photocoagulated, thus lowering the IOP more effectively than standard ECP [14,15]. The method of ciliary process photocoagulation in the current study was similar to that of ECP-plus; each ciliary process and the intervening space between processes were photocoagulated between a 180° and 270° circumference of the ciliary body, via a flat partial incision approach.

The main difference between the current method and ECP-plus was the method of ciliary process visualization. In combination with phacoemulsification for cataracts, we were able to observe the ciliary body in the pupil region under a microscope by pressing the eyeball. ECP typically requires a complete set of specialized equipment, including an 810 nm semiconductor laser, monitor, light source, and three-in-one 18–20G imaging probes. In contrast, the photocoagulation of ciliary processes under direct vision is simple and convenient. It can be widely performed using an argon or 532 nm semiconductor laser, without equipment restrictions.

As mentioned above, the range of photocoagulation is the first 2/3 of the ciliary process in standard ECP; therefore, 360° full photocoagulation of the ciliary process rarely results in postoperative hypotension and atrophy of the eye [16,17]. However, treatment with ECP-plus can lead to serious complications, such as postoperative hypotension and ocular atrophy [15]. Therefore, according to the principle of “less is better than more,” we set different photocoagulation ranges according to the preoperative IOP. The results of this case series showed that these photocoagulation ranges could effectively control the IOP and avoid the incidence of postoperative hypo-ocular pressure. Furthermore, we attempted a range of photocoagulation grading, but there was no correlation between the range of photocoagulation and the preoperative to postoperative change in IOP at the final examination. This may be related to the regeneration and migration of the remaining nonpigmented epithelium over time. Nevertheless, we consider the photocoagulation range from 180° to 270° as relatively safe. The photocoagulation volume should show abrupt whitening and collapse, with shrinkage of the ciliary process after photocoagulation. For different patients, different designs in terms of photocoagulation volume, photocoagulation range, and expected antihypertensive target are required.

On Kaplan–Meier survival analysis, the success rates were 80.0% and 77.1% at 6 months and 1 year postoperatively, respectively. These results are similar to the reported success rates for ECP-plus treatment of refractory glaucoma (81% and 78% at 6 months and 12 months postoperatively, respectively) [15]. In four eyes, the IOP was higher than the preoperative level on the first day postoperatively. However, after active anti-inflammatory treatment, the IOP rapidly decreased to below the preoperative level. This spike in IOP in the early postoperative period may be related to inflammatory exudation and viscoelastic residue.

**Figure 4:**
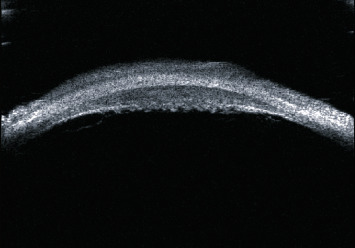
At 12 months postoperatively, ultrasonic biological microscopy shows complete atrophy of the ciliary processes at the photocoagulation site.

The success rate of standard ECP in the treatment of glaucoma in Asian patients has been reported to decrease significantly over time. This is because standard ECP can only destroy the first 2/3 of the ciliary process in ciliary epithelial cells; the remaining ciliary epithelial cells can regenerate and transition over time, and the function of aqueous fluid generation can be partially restored [18]. In contrast, the postoperative success rate of ECP-plus is relatively stable [15]. Consistent with this, the postoperative success rate did not significantly decrease with time in current case series. Postoperative ultrasonic biological microscopy examination also revealed that all ciliary processes were completely atrophied after photocoagulation (Figure 4). Therefore, the key to maintaining long-term stability in the IOP is to completely destroy the whole ciliary process, even the flat part of the ciliary body, and avoid the regeneration and migration of nonpigment epithelium.

The MD at the last follow-up postoperatively was reduced compared to that preoperatively, which may be related to the elimination of diffuse visual field defect caused by cataract. The RNFLT at the last follow-up postoperatively was reduced compared to that preoperatively. Considering the high IOP before surgery, the parapapillary RNFLT may form an edema. In the postoperative follow-up, although the IOP was normally controlled, the RNFLT still showed a thinning trend. In addition, decreased circulation perfusion of the RNFLT at high IOP, ischemia and hypoxia, and ischemia-reperfusion injury after decreased IOP are other factors causing thinning of the RNFLT.

In this case series, cataract phacoemulsification was performed simultaneously. Lens phacoemulsification can reduce the IOP in both normal and glaucomatous eyes [12]; however, IOP reduction is relatively minor in ECP combined surgery [16]. All patients in this case series had advanced PACG and mainly required pain relief and eyeball retention, with little expectation of visual function recovery. Accordingly, the patients underwent this destructive treatment. Although the postoperative IOP control with this surgical method was ideal, whether it can be promoted and applied to patients with good residual visual function and high requirements for retained visual function requires more case accumulation and further discussion.

## 5. Conclusions

This retrospective analysis of a small number of cases showed the short-term and medium-term efficacy of direct vision cyclo-body photocoagulation for the treatment of PACG. Cyclophotocoagulation under microscopic direct vision combined with phacoemulsification can be performed safely for the management of PACG. Evaluation of the long-term efficacy requires further observation. Since this study was retrospective in nature, with few cases, no controls, and a short follow-up, further studies with a larger sample size and longer follow-up, possibly randomized controlled trials, need to be conducted to understand the effectiveness of this surgical approach in such a blinding condition.

## Figures and Tables

**Table 1:** Absolute value of the MD and RNFLT before and after the operation.

Time	*N*	Absolute value of MD (dB)	RNFLT (*μ*m)
Preoperative	35	20.25 ± 6.11	69.89 ± 15.10
Last follow-up	35	19.26 ± 6.15	67.34 ± 14.76
Evaluation value		*Z* = −3.35	*Z* = −4.56
*P* value		*P*=0.001	*P* < 0.001

## Data Availability

The datasets generated and/or analyzed during the current study are not publicly available because of privacy concerns but are available from the corresponding author upon reasonable request.
